# Sympathetic cooling of positrons to cryogenic temperatures for antihydrogen production

**DOI:** 10.1038/s41467-021-26086-1

**Published:** 2021-10-22

**Authors:** C. J. Baker, W. Bertsche, A. Capra, C. L. Cesar, M. Charlton, A. Cridland Mathad, S. Eriksson, A. Evans, N. Evetts, S. Fabbri, J. Fajans, T. Friesen, M. C. Fujiwara, P. Grandemange, P. Granum, J. S. Hangst, M. E. Hayden, D. Hodgkinson, C. A. Isaac, M. A. Johnson, J. M. Jones, S. A. Jones, S. Jonsell, L. Kurchaninov, N. Madsen, D. Maxwell, J. T. K. McKenna, S. Menary, T. Momose, P. Mullan, K. Olchanski, A. Olin, J. Peszka, A. Powell, P. Pusa, C. Ø. Rasmussen, F. Robicheaux, R. L. Sacramento, M. Sameed, E. Sarid, D. M. Silveira, G. Stutter, C. So, T. D. Tharp, R. I. Thompson, D. P. van der Werf, J. S. Wurtele

**Affiliations:** 1 Department of Physics, College of Science, Swansea University, Swansea, SA2 8PP UK; 2 School of Physics and Astronomy, University of Manchester, Manchester, M12 9PL UK; 3 Cockcroft Institute, Sci-Tech Daresbury, Warrington, WA4 4AD UK; 4 TRIUMF, 4004 Wesbrook Mall, Vancouver, BC V6T 2A3 Canada; 5 Instituto de Fisica, Universidade Federal do Rio de Janeiro, Rio de Janeiro, 21941-972 Brazil; 6 Department of Physics and Astronomy, University of Calgary, Calgary, AB T2N 1N4 Canada; 7 Department of Physics and Astronomy, University of British Columbia, Vancouver, BC V6T 1Z1 Canada; 8 Department of Physics, University of California at Berkeley, Berkeley, CA 94720-7300 USA; 9 Department of Physics and Astronomy, Aarhus University, DK-8000 Aarhus C, Denmark; 10 Department of Physics, Simon Fraser University, Burnaby, BC V5A 1S6 Canada; 11 Department of Physics, Stockholm University, SE-10691 Stockholm, Sweden; 12 Department of Physics and Astronomy, York University, Toronto, ON M3J 1P3 Canada; 13 Department of Physics, University of Liverpool, Liverpool, L69 7ZE UK; 14 Experimental Physics Department, CERN, Geneva, 1211 Switzerland; 15 Department of Physics and Astronomy, Purdue University, West Lafayette, IN 47907 USA; 16 Soreq NRC, 81800 Yavne, Israel; 17 Department of Physics, Ben Gurion University, 8410501 Beer Sheva, Israel; 18 Physics Department, Marquette University, P.O. Box 1881, Milwaukee, WI 53201-1881 USA

**Keywords:** Optical manipulation and tweezers, Atomic and molecular interactions with photons, Exotic atoms and molecules, Plasma physics

## Abstract

The positron, the antiparticle of the electron, predicted by Dirac in 1931 and discovered by Anderson in 1933, plays a key role in many scientific and everyday endeavours. Notably, the positron is a constituent of antihydrogen, the only long-lived neutral antimatter bound state that can currently be synthesized at low energy, presenting a prominent system for testing fundamental symmetries with high precision. Here, we report on the use of laser cooled Be^+^ ions to sympathetically cool a large and dense plasma of positrons to directly measured temperatures below 7 K in a Penning trap for antihydrogen synthesis. This will likely herald a significant increase in the amount of antihydrogen available for experimentation, thus facilitating further improvements in studies of fundamental symmetries.

## Abstract (web summary)

Positrons are key to the production of cold antihydrogen. Here the authors report the sympathetic cooling of positrons by interacting them with laser-cooled Be^+^ ions resulting in a three-fold reduction of the temperature of positrons for antihydrogen synthesis.

## Introduction

The positron (e^+^) plays a unique role in physics. As the most readily available antiparticle, it had a significant role in the development of relativistic quantum mechanics. Its discovery^1^ confirmed the landmark predictions made by Dirac^2^. Its availability from radioactive sources meant that it quickly entered the laboratory, where tools such as positron annihilation spectroscopy are used for studies of materials and defects^3,4^. Positrons also play a key role in medical physics, e.g., positron emission tomography can be used to study biological processes in vivo^5^. A positron can form positronium, a short-lived hydrogen-like bound state with an electron, with annihilation limiting the lifetime^6^. This system is a unique, purely leptonic bound state that can be used for tests of fundamental symmetries^7^ and is also vital in the study of materials. The key motivation for the study presented here is that positrons and antiprotons can form antihydrogen, either via charge exchange from positronium or via a three-body reaction^8–10^. Antihydrogen, which does not appear to occur naturally, is a powerful tool for studying fundamental physics^11^. As an example, we recently found that the 1*S*–2*S* transition energy in antihydrogen agrees with that in hydrogen to a level of 2 × 10^−12^, the most precise and accurate comparison of hydrogen and antihydrogen to date^12^.

**Fig. 1:**
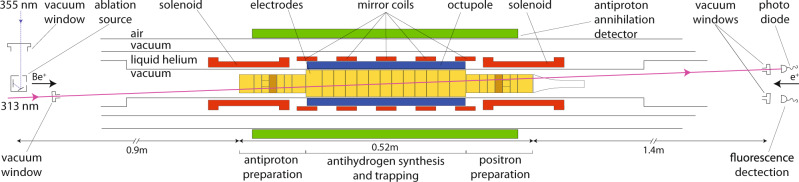
ALPHA trap setup with Be^+^ source and cooling laser. The main solenoid supplying a 1 T axial field throughout the center is not shown. The cylindrical electrodes (yellow/orange) can be individually charged to provide the axial electric fields for confinement. The central part is to scale. The short solenoids on either end can increase the axial field to 3 T in those regions. In typical operating conditions the field strength is homogenous to 1 part in 10^4^ across a cylindrical region of ø6 mm × 60 mm in the center^40^. The positron source and MCP/Phosphor assembly are to the right of the figure but not shown.

A cold, non-neutral plasma of positrons is a crucial ingredient in the only successfully demonstrated method for producing trappable antihydrogen. Synthesis occurs by merging a cold (<20 K) cloud of positrons with a cold cloud of antiprotons^13^. For merging, both species are confined in a cryogenic (~7 K) Penning-Malmberg trap, where radial confinement is secured by an axial magnetic field, and axial electric fields are used to confine and manipulate the charged particles along the length of the trap (Fig. 1). Experimental and theoretical evidence^13,14^ suggests that in the parameter range of current experiments, as opposed to previous ones^15^, antiprotons tend to thermally equilibrate with the positrons before antihydrogen is formed during this merging. A consequence is that the temperature of the nascent antihydrogen will be that of the positron plasma. Since, typically, only the tail of the thermal distribution of the formed antihydrogen has low enough energy to be trapped (≲0.5 K), achieving a lower positron temperature becomes the cornerstone of improving antihydrogen synthesis and trapping. Here, we report on the use of laser-cooled Be^+^ ions to sympathetically cool a positron plasma to cryogenic temperatures (~7 K) below those achievable by cyclotron cooling in our trap (~17 K). The resulting positron density and number are comparable to those used for antihydrogen synthesis, and our result thus paves the way for a significant increase in the number of trappable antihydrogen atoms. Although other techniques for cooling positrons to similarly low temperatures may be possible, such as resonant cyclotron cooling^16^, an advantage of the current method is that it required no modifications to the internal structure of the antihydrogen trap. It also has the advantage over a relatively simple technique such as evaporative cooling (EVC)^17^ in that particle numbers and densities can potentially be more carefully tuneable.

Be^+^ is the lowest mass ion that is laser-coolable from the ground state, and is therefore widely used for sympathetic cooling (e.g. refs. ^18,19^. Experiments in 2002 demonstrated sympathetic cooling of a small number of positrons (~2 × 10^3^) by laser-cooled Be^+^ ions in a 6 T Penning trap^20^ in which the ratio of ions to positrons was up to 500:1. For antihydrogen formation and capture in our apparatus, millions of positrons and lower magnetic fields (~1 T) are needed, and antiproton capture by Be^+^ ions needs to be minimized, such that a realistic Be^+^/e^+^ ratio has been estimated^21^ to be 1:10. Additionally, the geometry and cryogenic nature of the antihydrogen trap require that a Be^+^ ion source cannot be placed closer than about 1.2 m from the trap center, and that laser access is limited to be essentially on-axis and in one dimension (Fig. 1). These constraints currently also limit our solid angle for observing laser-induced fluorescence from trapped ions to be ~10^−6^ Sr. In this paper, we demonstrate, in a 1 T Penning–Malmberg trap for antihydrogen synthesis, by direct measurement of e^+^ temperatures and Be^+^ radial density distributions, sympathetic cooling of plasmas of millions of e^+^ to temperatures below 7 K in three dimensions using laser-cooled Be^+^ ions.

## Results

Figure 1 shows the layout of the experiment. The Be^+^ ions are produced by ablation of Be metal using a 355 nm, pulsed (6.3 ns) laser having ~75 µJ per pulse (fluence ~3 J cm^−2^)^22^. The resulting pulse of Be^+^ ions is dynamically captured in the left part of the trap system (Fig. 1) by using a blocking potential around the center of the trap and rapidly switching the potential on the first (left-most) electrode. Subsequently, the ions are moved to the axial central region, where there is optimal physical overlap with the 313 nm cooling laser. The system for laser cooling is a Toptica TA-FHG pro, frequency locked to a HighFinesse WS8-2 wavemeter. The laser beam is transported through air from a nearby laboratory and injected into the apparatus along a path that is at 2.3° to the central axis. Typically, 6 mW is injected. The beam radius (waist) is about 1.2 mm at the location of the ions. Fluorescence from the ions can be observed along a similar path that is azimuthally rotated by 180° with respect to the laser path (Fig. 1). Be^+^ is laser cooled using a laser beam, red detuned from the 2*s*^2^*S*_1/2_ (*m*_J_ = 1/2, *m*_I_ = 3/2) – 2*p*^2^*P*_3/2_ (*m*_J_ = 3/2, *m*_I_ = 3/2) transition. We rely on off-resonant optical pumping to transfer the population into the lower level. The magnetic field in the central region is ~1 T^23^. Positrons are transferred ballistically from a positron accumulator^24^ through a magnetic beamline and dynamically trapped in the positron preparation region (Fig. 1). After selecting the number of positrons^25^, we merge them with the Be^+^ ions, and the mixture is subsequently compressed using the rotating wall (RW) technique^26^, similar to the procedures we have developed for antiproton/electron mixtures^27^. Compression occurs in the positron preparation section, where an azimuthally split electrode is located. For improved compression, the axial B-field is first increased to 3 T, using a short solenoid (Fig. 1). Immediately following this step the Be^+^ and positron plasmas have a very good radial overlap, with a typical radius of around 0.6 mm. The mixture is subsequently moved to the central region where the Be^+^ can be laser-cooled. Following an experiment, the mixture can be ejected onto a microchannel plate/phosphor (MCP/phosphor) imaging detector that can also be used as a Faraday Cup. This allows measurement of the radial distribution of the plasmas^28^, their axial temperature^29^, or their total charge. The axial temperature is extracted by assuming that the cloud is in thermal equilibrium such that the initially released particles originate from the exponential tail of a Boltzmann distribution^17,29^. This also works when the plasma is a mixture of Be^+^ and e^+^, since the positron-induced signal on the MCP is more than an order of magnitude larger than that of the Be^+^, and the Be^+^ strikes the MCP later due to their larger mass. The axial temperature diagnostic suffers from systematic effects that include plasma cooling as the positron plasma expands in the process of being released, possible radial variation in the length and temperature of the plasma, and systematic errors in fitting the history of the released positrons to the theoretically expected curve. As we are not concerned with absolute comparisons here, we ensure relative consistency by always using the same potential well manipulations for the measurements. The errors on the positron temperatures quoted here are thus based on statistical variations only. The systematic effects may be a large as 50% on the *absolute* temperature determination, though particle-in-cell simulations for our parameter regime find that they are <10–15%^17,29,30^.

**Fig. 2:**
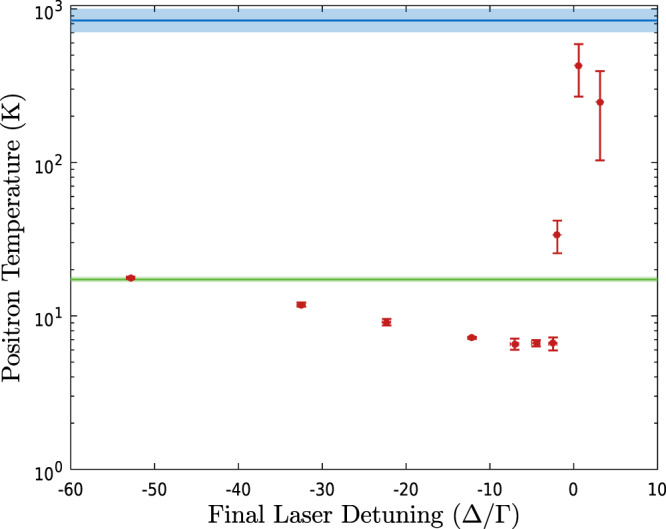
Axial e^+^ temperature in a mixed Be^+^/e^+^ plasma as a function of the final detuning (Δ) of the Be^+^ cooling laser. The mixed plasma consisted of 2.6 × 10^6^ e^+^, with an initial radius and density of 0.64 mm and 3 × 10^8^ cm^−3^, respectively, and 3.8 ± 0.1 × 10^5^ Be^+^ ions. The laser frequency was swept from an initial detuning of −104Γ to the final laser detuning in ~40 s. The error bars give the standard error of multiple measurements at each setting. The green line shows the e^+^ temperature in the absence of any Be^+^ ions (17.3 ± 0.5 K), and the blue line shows the e^+^ temperature when the laser is blocked and the Be^+^ ions are not laser-cooled. The shading around these lines indicates the standard error.

In the first set of experiments, we prepared a sample of 2.6 × 10^6^ positrons with a radius (in 1 T) of 0.54 mm, representative of samples used in our antihydrogen experiments. This sample was merged with 3.8 × 10^5^ Be^+^. The Be^+^/e^+^ mixture was held in the central region in a single-electrode well having an on-axis depth of 70 V. In order to efficiently cool the whole population of Be^+^, the laser was initially detuned 104Γ below the cooling transition (Γ = 2*π* × 19.6 MHz is the natural linewidth of the transition), and then scanned in 40 s to a final detuning that was varied. The frequency scan time was optimized experimentally to obtain the lowest e^+^ temperatures. Figure 2 shows the temperature of the e^+^ plasma measured as a function of the final detuning. Between −7Γ and −2Γ, the e^+^ temperature reaches a minimum of 6.6 ± 0.5 K, which is a factor ~2.6 below that reported (using an identical temperature diagnostic) for e^+^ in the synthesis of antihydrogen. For comparison, a Doppler dominated width (HWHM) of 7Γ = 140 MHz (the detuning for the onset of the temperature minima) would correspond to a Be^+^ temperature of 1.5 K.

The measured minimum e^+^ temperature is lower with laser-cooled Be^+^ present than it is in the absence of either Be^+^ or the cooling laser, and the temperature depends on the laser detuning. Thus, Fig. 2 clearly shows a sympathetic cooling effect. However, the flat minimum, and the fact that the e^+^ temperature is higher than the expected ion temperature for these detunings, indicate that the sympathetic cooling saturates. We cannot exclude that we have reached the lower limit of our temperature diagnostic, but, as we discuss below, centrifugal separation of the two species at these low temperatures likely leads to the observed saturation. The temperatures extracted from those independent measurements are in agreement with those measured here. In addition, the temperature of the mixed plasma without laser cooling is significantly above those typical for e^+^ in our apparatus. We believe that the higher than expected temperatures can be attributed to increased heating due to the presence of Be^+^. In equilibrium, a strongly coupled, non-neutral plasma rotates rigidly around the axis of the trap with a constant frequency^31^. In a mixed species, non-neutral plasma the species centrifugally separate, with the more massive species accumulating at larger radius^32^. Ion plasmas are, due to their larger mass and lower axial bounce frequencies, more susceptible to field inhomogeneities and expand faster than similar lepton plasmas, the expansion being accompanied by heating (cf. ref. ^33^). In a mixed plasma, friction between the species drives the accompanying e^+^ expansion. We measured the different expansion rates in independent tests (see the “Methods” section) and found that both Be^+^-only and mixed samples expand radially almost two orders of magnitude faster than e^+^-only samples in our parameter range.

**Fig. 3:**
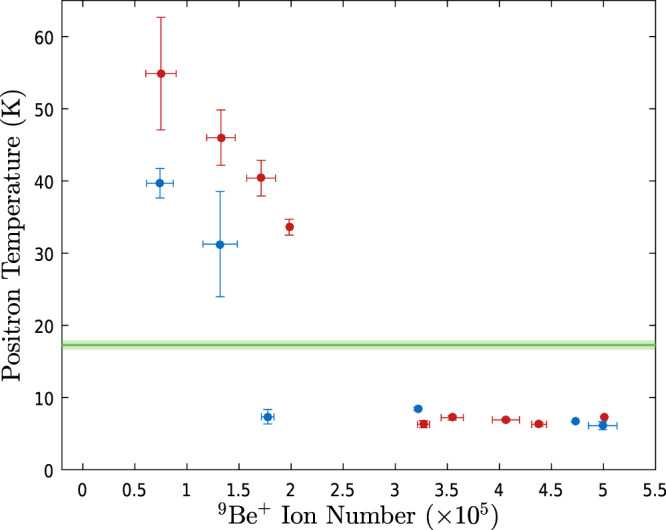
Positron temperature as a function of the number of Be^+^ ions used for sympathetic cooling. The ion number has been binned with the horizontal error bars representing the standard deviation of the mean ion number within each bin. The vertical error bars correspond to the standard error on the mean temperature within each bin. The ion number was varied by changing the number of ablation pulses used for loading, and also by splitting the ion plasma using axial potential manipulations. We used 1.4 × 10^6^ positrons (blue points) and 2.6 × 10^6^ positrons (red points) with initial densities of 1 × 10^8^ and 3 × 10^8^ cm^−3^, respectively. The cooling laser frequency was swept from an initial detuning of −129Γ to a final detuning of −7Γ in ~40 s for sympathetic cooling of the positrons. The positron temperature for 2.6 × 10^6^ positrons with no ions present (green line) is shown for comparison. The shading around this line indicates the standard error. Note that the positron-only temperature for 1.4 × 10^6^ positrons is consistent with this temperature.

As positrons are cooled through collisions with laser-cooled Be^+^ ions, increasing the Be^+^ to e^+^ number ratio could improve sympathetic cooling. To investigate this, we used a fixed final detuning of −7Γ while varying the Be^+^ number over a large range, at a fixed positron number. Figure 3 shows the final e^+^ temperature as a function of the number of Be^+^ ions for two different e^+^ numbers. What we observe is that in both cases, when the Be^+^ number is above a threshold the sympathetic cooling is efficient, yielding a low e^+^ temperature that is independent of the Be^+^ number and consistent with the lowest in Fig. 2. The threshold is roughly consistent with a Be^+^ to e^+^ number ratio of 1:10. When the Be^+^ number is too low we measure e^+^ temperatures above those observed without ions. The threshold behavior is consistent with the simulations in ref. ^21^, where it was determined that strong centrifugal separation at low temperatures caused diminishing returns of further ion number increases; additional ions end up at higher radii and do not contribute to cooling the e^+^. The higher than expected temperatures observed with too few Be^+^ indicates, as discussed previously, additional heating in the presence of ions.

**Fig. 4:**
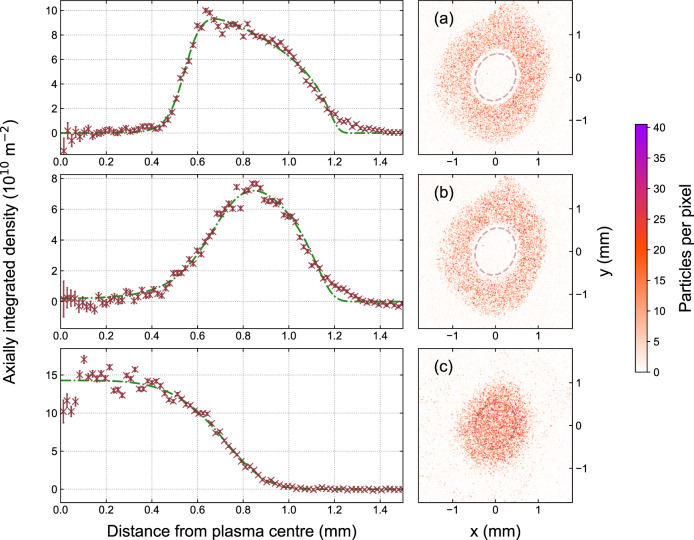
Be^+^ density profiles from originally mixed samples of Be^+^ and e^+^ with 2 × 10^5^Be^+^ and 1.2 × 10^5^e^+^. The images (right) show Be^+^ ejected to the MCP/Phosphor imaging assembly 5 µs after the ejection of e^+^ in the opposite direction immediately following the laser-cooling of the Be^+^. **a** Laser detuning −20Γ, **b** Detuning −36Γ, and **c** Detuning −128Γ. The measured longitudinal temperatures were **a** 7.1 ± 0.5 K, **b** 10.1 ± 0.2 K, and **c** 370 ± 100 K, respectively. To the left of each image is a plot of the extracted axially integrated radial density profile, over-laid with the best fit distribution from calculations assuming thermal equilibrium at the temperatures stated. The fit results were **a** 6.2 ± 0.6 K, **b** 19 ± 2 K and **c** 253 ± 54 K. The corresponding e^+^ densities were **a**, **b** 6.2 ± 0.1 × 10^7^ cm^−3^ and **c** 1.2 ± 0.2 × 10^8^ cm^−3^. The calculations were done using the N2DEC code in ref. ^34^. where thermal equilibrium of the two species is assumed. Note that due to the distortion in the imaging, the plasma image is elliptical; the extraction of the radial profile took this into account. Additionally, at higher radii (>1.25 mm) the images are distorted by stray electric fields near the MCP as well as physical aperture effects.

To investigate the Be^+^ radial distribution and thus the centrifugal separation, we first eject the e^+^ by lowering the confining potential in the direction opposite to the MCP for ~100 ns, short enough to prevent Be^+^ ions from escaping the trap. This is followed almost immediately (~5 µs) by the ejection of the Be^+^ towards the MCP for imaging. The delay is minimized to prevent the collapse of the hollow Be^+^ distribution^34^. We remove the e^+^ first because the e^+^ signal overwhelms that of the Be^+^ on the MCP. For these measurements, we use 2 × 10^5^ Be^+^ and 1.2 × 10^5^ e^+^. Fewer particles were used in this study to ensure that we could correctly image the entire radial extent of the plasma on the MCP after laser cooling. Figure 4 shows MCP images and extracted radial distributions of the Be^+^ plasmas ejected in this way for three different final laser detunings. We fitted the measured radial distributions to distributions extracted from self-consistent e^+^/Be^+^ thermal equilibrium calculations using the N2DEC code from ref. ^34^. We find, for the coldest plasma, a temperature of 6.2 ± 0.6 K (Fig. 4a), in good agreement with the measured longitudinal e^+^ temperature of 7.1 ± 0.5 K. At these temperatures and our experimental densities the e^+^ collision rates are of order kHz, ensuring thermal equilibrium on the timescale of the experiment. We thus conclude that we have sympathetically cooled the e^+^ plasmas longitudinal and transverse motions to cryogenic temperatures. We also observe, as expected^21,32^, that the amount of separation of the two species increases as the temperature drops, thus reducing the inter-species interactions. While the positron numbers were lower in this part of the experiment, the good agreement with the equilibrium calculations means that centrifugal separation is likely also a key factor in causing the cooling saturation shown in Fig. 2.

**Fig. 5:**
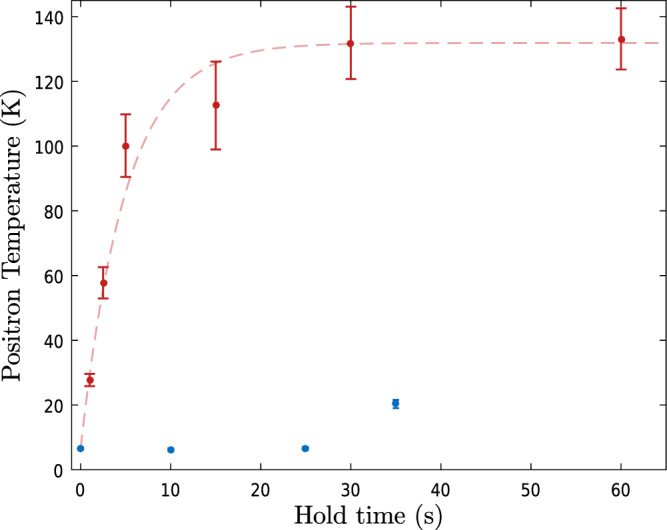
Evolution of the e^+^ temperature following the laser cooling frequency sweep. 1.4 × 10^6^ e^+^ and 4.7 ± 0.1 × 10^5^ Be^+^ ions were used. Turning the cooling laser off (red points) results in the e^+^ heating at a rate of ~25 K s^−1^, before their temperature saturates at around 125 K. If, however, the cooling laser is kept on at a fixed detuning of −7Γ, the final detuning in the frequency sweep, the e^+^ can be kept at 6.7 ± 0.3 K for over 20 s (blue points). The vertical error bars correspond to the standard error on the mean temperature for each hold time.

Our current fluorescence detection capability is limited to Be^+^ temperatures below ~500 mK and cannot be used to determine the temperature of Be^+^/e^+^ mixtures (see the “Methods” section). From fluorescence detection, we find a typical longitudinal temperature of 200 mK for 10^6^ laser-cooled Be^+^ ions in our system. This is well below the lowest temperature of our sympathetically cooled e^+^. To look for additional heating that may explain this, we examined the e^+^ temperature evolution in the mixture with or without sustained laser-cooling. The measurements are shown in Fig. 5, where we find that the heating rate is initially 25 K/s for the coldest sympathetically cooled e^+^. Such temperatures can be maintained for over 20 s when laser-cooling remains active, which is much longer than the typical 1 s used for antihydrogen formation in our experiment. In contrast, e^+^ prepared alone heat from an initial temperature of ~18 K at a rate below 1.5 K/s. The measured mixed plasma heating rate is similar to the rate used in simulations that achieved a low-temperature limit of ~5 K^21^, in good agreement with the limits observed experimentally. The e^+^ temperature increase observed after prolonged sympathetic cooling (Fig. 5) is likely due to radial expansion eventually reducing the Be^+^ overlap with the cooling laser.

We conclude that the observed low-temperature limit for sympathetically cooled e^+^ is given by the combination of additional heating caused by the presence of Be^+^ and the reduced sympathetic cooling as the species centrifugally separate at low temperatures. As discussed previously this heating is likely caused by the Be^+^ plasmas rapid expansion, and may, in part be overcome by, e.g., working in higher magnetic fields (like in ref. ^20^), or by using shorter potential wells. Higher fields would reduce the depth of the antihydrogen trap, but are only available in the positron and antiproton preparation sections of our trap, where laser access is foreseen but not currently available. Shorter potential wells can also (currently) only be achieved in the side sections.

Finally, we address the question of the method’s compatibility with the inhomogeneous magnetic field of the antihydrogen atom trap. To remain compatible the plasmas must be kept radially small at all times to avoid adverse effects from the transverse octupole magnetic fields of the atom trap^35,36^. To investigate this, we sympathetically cooled 10^5^e^+^ with a radial extent of 0.6 mm using 10^5^ Be^+^ to a temperature of 6.8 ± 0.5 K in the magnetic field used for trapping antihydrogen, thereby demonstrating the feasibility. Unfortunately, the setup currently does not allow laser-cooling while applying the RW compression. We were, therefore, unable to perform this test on larger e^+^ samples, as their radii were too large to avoid the detrimental effects of the non-uniform magnetic fields. Modification of the apparatus to allow laser-cooling during RW and several other improvements are underway to make the system fully compatible with antihydrogen accumulation once antiprotons return to the Antiproton Decelerator at CERN in 2021.

In conclusion, we have demonstrated, by direct measurement of e^+^ temperatures and Be^+^ radial density distributions, sympathetic cooling of plasmas of millions of e^+^ to temperatures below 7 K in three dimensions using laser-cooled Be^+^ ions. The density and size of the e^+^ plasmas as well as the magnetic fields used are commensurate with those used for antihydrogen synthesis and trapping. Efficient sympathetic cooling was also demonstrated in the inhomogeneous fields used to confine antihydrogen. The lowest temperatures are almost a factor of three lower than the lowest currently used for antihydrogen formation. The corresponding decrease in the expected temperature of the synthesized antihydrogen leads us to expect an improvement in the amount of trapped antihydrogen per mixing attempt of up to a factor of five. Note that we do not expect much increase in the absolute amount of antihydrogen formed since even at standard positron temperatures (~17 K) about 60% of antiprotons form antihydrogen atoms. The expected increase in the antihydrogen trapping rate paves the way for faster and more precise measurements of antihydrogen.

## Methods

### Plasma parameter control and measurement

Positrons are accumulated from a radioactive source-based beam using a Surko-type three-stage accumulator, where a buffer gas provides the cooling^37^. Subsequently, they are transferred ballistically to the main apparatus through a magnetic beamline, where they are captured using a fast switching gate electrode. The transfer efficiency was around 16% in these experiments, and the number of positrons captured typically fluctuates by more than 10%. To reduce these fluctuations to below 1% we used a combination of a strong drive regime rotating wall (SDR) and EVC that we have developed previously^25^. The remaining positrons then cool through the emission of cyclotron radiation and an adiabatic expansion cooling step to a temperature of ~17 K. We have not carefully characterized what sets the lower limit on this value, but we expect that it is mainly due to electronic noise. Also, although we do not directly measure the energy distribution of the positrons transverse to the magnetic field, we expect both the longitudinal and transverse motions to thermalize through collisions in the density and temperature regime that we are operating in^38^.

**Fig. 6:**
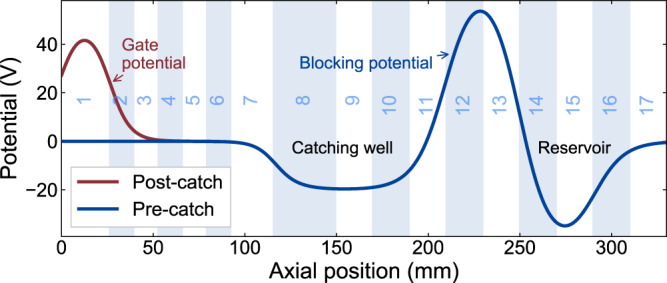
Potentials on-axis for Be^+^ capture and stacking. Shaded and numbered regions represent electrode positions. Ions enter from the left into the blue potential. Subsequently, the gate electrode (E1) is raised to 40 V (red potential) to capture them. Ions are then moved to the reservoir where they are combined with previous loads and laser-cooled until the desired number of stacks have been accumulated.

Beryllium is loaded in a similar fashion to the positrons in the opposite end of the apparatus (Fig. 6). Be^+^ ions are emitted directly from our ablation target^22^ located on the axis of the setup (Fig. 1) and subsequently after they have had time to arrive in the main trap system, dynamically captured using the gate electrode. The mean energy of the Be^+^ from ablation is around 20 eV for our ablation laser fluence of 3 J cm^−2^. A single capture results in 10^5^–10^6^ ions. To better control the number of ions we capture multiple batches by repeating the above process several times. The previously captured Be^+^ ions are laser-cooled with a fixed detuning of −129Γ between batches by moving them to the center of the trap system (cf. Fig. 6). The large detuning reflects the large energy spread of the captured ions (~20 eV). The number of ions captured in this way fluctuates about 30% between identical runs.

The radial size of the plasmas is controlled by the application of a strong-drive regime RW where a rotating dipolar electric field exerts a torque on the plasma causing it to rotate faster or slower and thereby compress or expand^39^. In our setup, we have two locations where we have the necessary azimuthally split electrodes, one in the antiproton capture section and one in the positron capture section as indicated in Fig. 1. The principle of the RW requires there to be a slight slip between the rotating electric dipole field and the plasmas self-rotation to transfer torque. This slip results in heating of the plasma, and the RW can therefore only be applied effectively for a short while without cooling. Positrons cool through the emission of cyclotron radiation in our 1–3 T fields, but for Be^+^ ions we need laser-cooling. As the laser only overlaps in the central part of the apparatus we implemented an arrested procedure where we move the Be^+^ plasma between a laser-cooling region, where it is cooled with a fixed detuning at −129Γ, and an RW region multiple times until the desired radial extent is achieved.

**Fig. 7:**
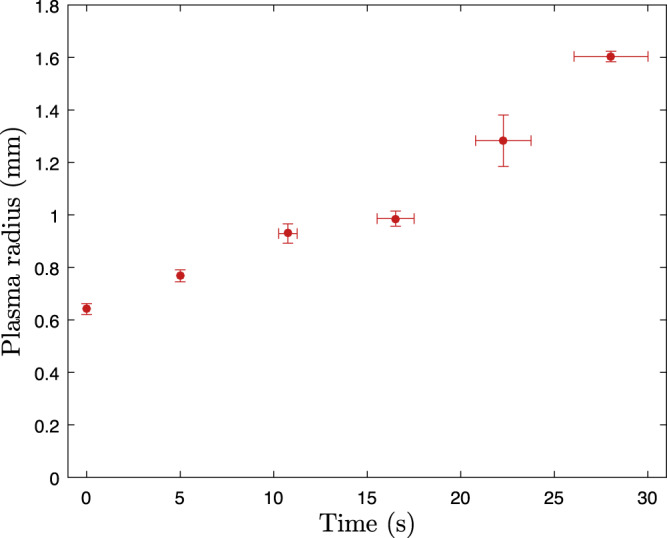
Evolution of the radius of the positron plasma as a function of time during the laser cooling frequency sweep. The plasma was a mixture of 2.6 × 10^6^ e^+^ and ~4–5 × 10^5^ Be^+^ ions. Time zero is when the frequency sweep begins. The total sweep time we use to sympathetically cool the positrons to their lowest temperatures is 40 s. It is not possible to measure plasma radii greater than around 1.6 mm with our current MCP imaging diagnostic. The error corresponds to the standard error on the mean radius measured (vertical bars) and the laser-cooling time (horizontal).

**Fig. 8:**
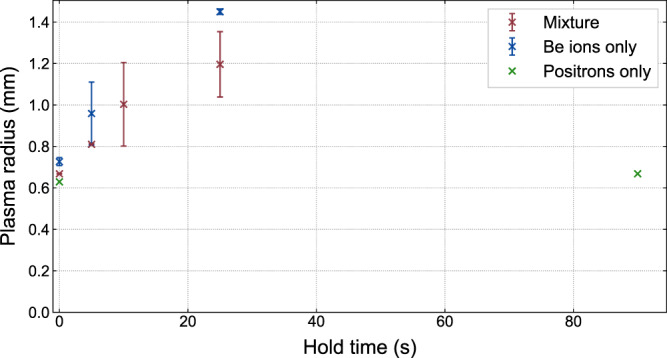
Evolution of the radius of three different plasmas as a function of time without laser-cooling in a 1 T field. The mixture (red) consists of 2.6 × 10^6^ e^+^ and 4 × 10^5^ Be^+^. The Be^+^ only plasmas contained 4 × 10^5^ Be^+^. The positrons only plasma contained 2.6 × 10^6^ e^+^. The vertical error bars correspond to the standard error on the mean radius measured at each hold time.

Figure 7 shows measurements of the radial size of the positrons in a mixed plasma during laser-cooling. As discussed in the paper, the mixed plasma also expands during cooling. As shown in Fig. 8 this expansion is not observed when only e^+^ are present, but it is also present for a pure Be^+^-only sample. The total time needed for cooling is, therefore, an important factor in determining the final radial extent of the positron plasma and therefore its density.

As the ion to positron ratio is important for the sympathetic cooling, and only the positron number is stable, we implemented a technique whereby we could measure both the positron temperature and the Be^+^ number in each run (except for the special runs where we image the Be^+^ plasma to measure the centrifugal separation, as explained in the main part of paper). When measuring axial temperatures by slowly ejecting particles from a well the temperature information is in the initial exponential tail of particles being ejected^29^. There is thus no reason to eject all the e^+^ to measure the temperature. Since Be^+^ is centrifugally separated from the e^+^ and the potential is the lowest in the center, Be^+^ will not be ejected if the well depth is only reduced slightly below the threshold of the first particles escaping. We, therefore, perform the temperature measurement using a partial ejection by reducing the well to about 60% of the potential where particles first appear (recall, that the e^+^ number is reproducible to 1%). Subsequently, we close the well and eject the remaining positrons using a 100 ns opening of the well, which is too short for any Be^+^ to escape. Finally, we eject the Be^+^ ions to the MCP for counting (at this point any original radial extent information will be lost).

### Laser setup for ablation and cooling

**Fig. 9:**
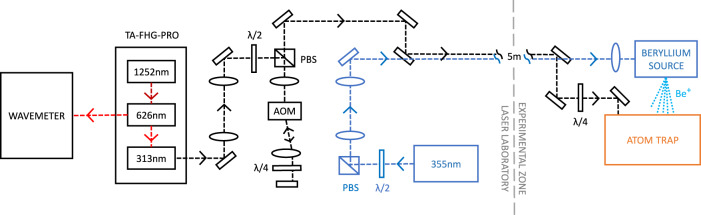
Laser-setup for ablation (blue) and laser-cooling (black). The TA-FHG PRO from Toptica generates 313 nm by two doubling stages of a 1252 nm amplified diode laser. The second harmonic light at 626 nm is sent to a wavemeter for frequency control (see below). The double-pass AOM setup is used to sweep the laser for fluorescence-based detection (the detection geometry was indicated in Fig. 1). The polarization beam splitters (PBS) for both lasers serve, through changes of polarization to control the power delivered to the experiment. Both lasers are passed through a periscope setup from the laser room to the experiment. The quarter-wave plate on the 313 nm path before it enters the Atom Trap serves to tune the polarization.

Two separate laser systems were used for these experiments. Both were located in a laser laboratory adjacent to the main experimental setup. Figure 9 shows a schematic of the setup of the two laser systems. We used a pulsed Nd:YAG laser (Quantel Ultra 20) operating on the 3rd harmonic at 355 nm delivering 6.3 ns long pulses with ~75 µJ per pulse. The pulses are focussed with a *f* = 25 cm convex lens, resulting in a fluence of ~3 J cm^−2^ on the beryllium metal target to generate Be^+^ ions via laser ablation. The ion source is aligned with the beam axis of the main apparatus using a linear translator (cf. Fig. 1). The Toptica TA FHG-PRO system generates 313 nm light for laser cooling by twice doubling the light from a 1252 nm amplified laser diode. A fraction of the 626 nm light from the first doubling stage is sent to a HighFinesse WS8-2 wavemeter for locking the laser frequency. The 313 nm light is circularly polarized with a quarter-wave plate before entering the apparatus.

### Fluorescence detection

**Fig. 10:**
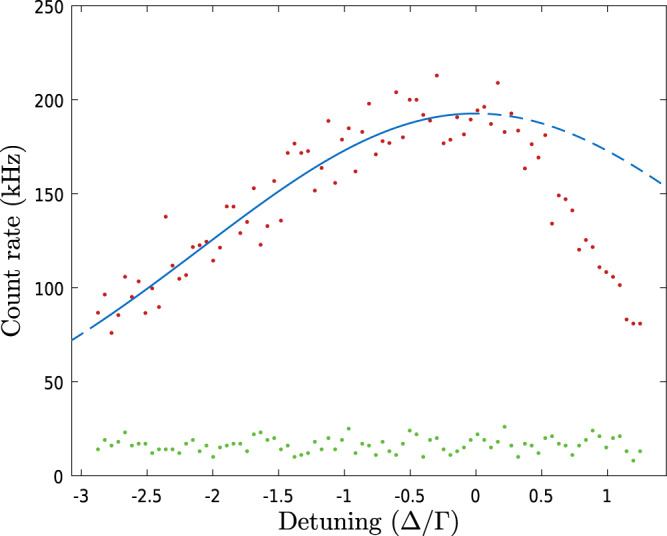
Fluorescence signal from a pure Be^+^ plasma as the frequency of the cooling laser is scanned across the laser cooling transition. The background-corrected signal (red points) has been fit with a Voigt function (blue solid line) on the left-hand side of the peak. It is clear that the function fits poorly if it is continued onto the right-hand side of the peak (blue dashed line) where the fluorescence signal drops off rapidly. This sharp decay in signal is attributed to the heating of the ions when the laser detuning is on the blue side of the resonance. The Voigt profile corresponds to an ion temperature of ~150 mK. The background signal on the detector when no ions are loaded, which comes mainly from the scatter of the laser beam within the apparatus, is shown for reference (green points).

As mentioned in the article the solid angle for observing fluorescing ions is currently limited to about 10^−6^ Sr. In order to measure a Be^+^ temperature we can use the AOM (see Fig. 9) to chirp the laser light across the resonance and measure the Doppler-broadened linewidth and thereby the temperature. The AOM has a sweeping range of 0–80 MHz (~4Γ). Taken together this allows us only to measure the temperature of Be^+^ samples colder than ~500 mK. Figure 10 shows an example of a measurement of ~1M Be^+^ ions laser-cooled with a final detuning of −3Γ. To extract the Be^+^ temperature we fit the left-hand side of the fluorescence peak with a Voigt function. The asymmetry resulting from the heating of the ions when the laser is on the high-frequency side of the resonance means that the right-hand side of the peak is ignored in the analysis. In order to extract a temperature, the Voigt function is deconvolved into its Lorentzian and Gaussian components. From the Gaussian full width at half maximum, the ion temperature can then be calculated. Due to limitations in the AOM frequency range and the small solid angle we cannot currently conduct fluorescence-based temperature diagnostics of Be^+^ in mixed samples of Be^+^ and e^+^ where the Be^+^ number is much lower and the temperature higher than in the example measurement in Fig. 10.

### Be^+^ laser frequency control

The frequency of the 313 nm cooling laser is regulated via modulation of the master laser diode current in a PID loop. The modulation signal is provided by a wavemeter which is continuously measuring the frequency of the second harmonic (at 626 nm) of the master laser. The wavemeter is calibrated at the beginning of every experimental run using a stable He–Ne laser as a frequency reference. When sweeping the frequency to laser cool the Be^+^ ions, and sympathetically cool positrons, we discretely step the frequency setpoint such that the laser frequency changes at an average rate of ~70 MHz/s between setpoints. Discrete steps in the setpoint were used to ensure that the PID loop changed the frequency of the laser smoothly, and slowly enough such that the ions are continuously cooled from their initial high temperature. Unfortunately, due to hardware resources shared with other aspects of the ALPHA apparatus, the wavemeter was not fully integrated with the control system managing sympathetic cooling, which resulted in latency and jitter when requesting changes to the laser frequency. The relatively large horizontal error bars in Fig. 7 are a result of this jitter. Another consequence of this uncertainty is that we could not use this method of frequency modulation when measuring fluorescence spectra. Faster, deterministic frequency sweeps were performed when measuring the Doppler broadened fluorescence spectra of the Be^+^ ions. In this case, we used an AOM to sweep the frequency of the light by 80 MHz in 80 ms (Fig. 9). By performing several fluorescence measurements over different days, we have determined that the location of the laser-cooling resonance is consistent to within 9 MHz (standard deviation), corresponding to ~Γ/2. The apparent fluctuation in the resonance location is most likely due to a combination of the imperfect laser frequency lock, the calibration and precision of the wavemeter, and our ability to locate the resonance of the strongly asymmetric line shape.

## Data Availability

The small datasets generated during and/or analyzed during the current study are available from N.M. and J.S.H. (niels.madsen@cern.ch, jeffrey.hangst@cern.ch) on reasonable request.

## Code availability

The code N2DEC used for the equilibrium solutions is available from N.M. and J.S.H. (niels.madsen@cern.ch, jeffrey.hangst@cern.ch) on reasonable request.

## References

[1] Anderson CD (1933). The positive electron. Phys. Rev..

[2] Dirac PAM (1930). A theory of electrons and protons. Proc. R. Soc. A.

[3] Tuomisto F, Makkonen I (2013). Defect identification in semiconductors with positron annihilation: experiment and theory. Rev. Mod. Phys..

[4] Hugenschmidt C (2016). Positrons in surface physics. Surf. Sci. Rep..

[5] Bailey, D. L. et al. *Positron Emission Tomography* (Springer London Ltd, 2005).

[6] Deutsch M (1951). Evidence for the formation of positronium in gases. Phys. Rev..

[7] Karshenboim SG (2005). Precision physics of simple atoms: QED tests, nuclear structure and fundamental constants. Phys. Rep..

[8] Baur G (1996). Production of antihydrogen. Phys. Lett. B.

[9] Amoretti M (2002). Production and detection of cold antihydrogen atoms. Nature.

[10] Storry CH (2004). First laser-controlled antihydrogen production. Phys. Rev. Lett..

[11] Kostelecky VA, Vargas AJ (2015). Lorentz and CPT tests with hydrogen, antihydrogen and related systems. Phys. Rev. D.

[12] Ahmadi M (2018). Characterization of the 1*S*–2*S* transition in antihydrogen. Nature.

[13] Ahmadi M (2017). Antihydrogen accumulation for fundamental symmetry tests. Nat. Commun..

[14] Jonsell S, Charlton M (2018). On the formation of trappable antihydrogen. N. J. Phys..

[15] Madsen N (2005). Spatial distribution of cold antihydrogen formation. Phys. Rev. Lett..

[16] Hunter ED (2018). Low magnetic field cooling of lepton plasmas via cyclotron-cavity resonance. Phys. Plasmas.

[17] Andresen GB (2010). Evaporative cooling of antiprotons to cryogenic temperatures. Phys. Rev. Lett..

[18] Schmöger L (2015). Coulomb crystallization of highly charged ions. Science.

[19] Barrett MD (2003). Sympathetic cooling of ^9^Be^+^ and ^24^Mg^+^ for quantum logic. Phys. Rev. A.

[20] Jelenkovic BM (2003). Sympathetically cooled and compressed positron plasma. Phys. Rev. A.

[21] Madsen N, Robicheaux F, Jonsell S (2014). Antihydrogen trapping assisted by sympathetically cooled positrons. N. J. Phys..

[22] Sameed M, Maxwell D, Madsen N (2020). Ion generation and loading of a Penning trap using pulsed laser ablation. N. J. Phys..

[23] Amole C (2014). In situ electromagnetic field diagnostics with an electron plasma in a Penning-Malmberg trap. N. J. Phys..

[24] Amole C (2014). The ALPHA antihydrogen trapping apparatus. Nucl. Instrum. Methods A.

[25] Ahmadi M (2018). Enhanced control and reproducibility of non-neutral plasmas. Phys. Rev. Lett..

[26] Huang XP (1997). Steady-state confinement of non-neutral plasmas by rotating electric fields. Phys. Rev. Lett..

[27] Andresen GB (2008). Compression of antiproton clouds for antihydrogen trapping. Phys. Rev. Lett..

[28] Andresen GB (2009). Antiproton, positron and electron imaging with a microchannel plate/phosphor detector. Rev. Sci. Instrum..

[29] Eggelston DL (1992). Parallel energy analyzer for pure electron plasma devices. Phys. Fluids B.

[30] Hunter ED (2020). Plasma temperature measurement with a silicon photomultiplier (SiPM). Rev. Sci. Instrum..

[31] Dubin DHE, O’Neil TM (1999). Trapped nonneutral plasmas, liquids, and crystals (the thermal equilibrium states). Rev. Mod. Phys..

[32] O’Neil TM (1981). Centrifugal separation of a multispecies pure ion plasma. Phys. Fluids.

[33] Kabantsev AA, Yu JH, Lynch RB, Driscoll CF (2003). Trapped particles and asymmetry-induced transport. Phys. Plasmas.

[34] Andresen GB (2011). Centrifugal separation and equilibration dynamics in an electron-antiproton plasma. Phys. Rev. Lett..

[35] Fajans J (2005). Effects of extreme magnetic quadrupole fields on penning traps and the consequences for antihydrogen trapping. Phys. Rev. Lett..

[36] Butler, E. *Antihydrogen Formation, Dynamics and Trapping, Sec 3.5.* PhD thesis, Swansea University (2011).

[37] Murphy TJ, Surko CM (1992). Positron trapping in an electrostatic well by inelastic collisions with nitrogen molecures. Phys. Rev. A.

[38] Glinsky ME (1992). Collisional equipartition rate for a magnetized pure electron plasma. Phys. Fluids B.

[39] Danielson JR, Surko CM (2005). Torque-balanced high-density steady states of single-component plasmas. Phys. Rev. Lett..

[40] Ahmadi M (2017). Observation of the 1*S*–2*S* transition in trapped antihydrogen. Nature.

